# The ballistic performance of bone when impacted by fragments

**DOI:** 10.1007/s00414-020-02299-9

**Published:** 2020-05-02

**Authors:** A. J. Caister, D. J. Carr, P. D. Campbell, F. Brock, J. Breeze

**Affiliations:** 1 Caister Consultancy Ltd, Winchester, SO23 9HX UK; 2 AWE, Aldermaston, Reading, RG7 4PR UK; 3 Defence and Security Accelerator, Porton Down, Wiltshire, SP4 0JQ UK; 4 Centre for Defence Engineering, Defence Academy of the United Kingdom, Cranfield University, Shrivenham, Oxon SN6 8LA UK; 5 Department of Life Sciences, Natural History Museum, South Kensington, London, SW7 5BD UK; 6 Cranfield Forensic Institute, Defence Academy of the United Kingdom, Cranfield University, Shrivenham, Oxon SN6 8LA UK; 7 Royal Centre for Defence Medicine, University Hospitals Birmingham, Birmingham, B15 2TH UK

**Keywords:** Swine ribs, Armour design, Wound ballistics, Micro-CT

## Abstract

Physical models are required to generate the underlying algorithms that populate computer simulations of the effects of explosive fragmenting devices. These models and simulations are used for understanding weapon performance, designing buildings and optimising personal protective equipment. Previous experimental work has investigated the performance of skin and muscle when subjected to fragmentation threats, but limited evidence exists for the performance of bone when impacted by fragments. In the current work, ballistic testing was conducted using two types of internationally recognised steel fragment simulating projectiles (FSPs): (i) 5.5 mm diameter (0.68 g) ball bearing (BBs) and (ii) 1.10 g chisel nosed (CN). These projectiles were fired at isolated swine ribs at impact velocities between 99 and 1265 m/s. Impact events were recorded using a high-speed camera. Selected specimens were analysed post-impact with plain x-radiographs and micro-CT scanning to determine damage to the bone architecture. Bones were perforated with a kinetic energy density (KED) as low as 0.14 J/mm^2^. Energy transfer to the bone was greater for the CN FSPs, resulting in increased bone damage and the production of secondary bone fragments. The manner in which the bones failed with faster velocity impacts (> 551 m/s; KED > 6.44 J/mm^2^) was analogous to the behaviour of a brittle material. Slower velocity impacts (< 323 m/s; KED < 1.49 J/mm^2^) showed a transition in failure mode with the bone displaying the properties of an elastic, plastic and brittle material at various points during the impact. The study gives critical insight into how bone behaves under these circumstances.

## Introduction

Fragment-induced injury is a hazard faced by military personnel and civilians in modern combat and in domestic terrorist environments [e.g. 1–4]. The optimisation of personal armour design and the understanding of medical techniques needed to treat ballistic injuries can benefit from injury models. Traditionally, these have been physical models using stimulants such as gelatine, post mortem human subject (PMHS) tissue and animal surrogates [5, 6]. Many modern injury models use a computerised representation of human anatomy to predict how it may respond to a ballistic threat. Such models can be advantageous as expensive test facilities are not required once the dataset has been established. However, such computational models require an accurate understanding of the interaction between projectile parameters (e.g. material, mass, velocity, density, shape, deformation due to interaction with the target) and the severity of tissue damage to make robust and accurate injury predictions. Therefore, understanding the ballistic performance of the various tissues for use in computational models is vital to their success.

PMHS tissue is difficult to obtain and demonstrates the variability seen among all cadaveric tissue specimens; there are also ethical and legal complications. Swine tissue is one of the most common surrogates used in testing due to its wide availability and similarities between some human and pig body sections and bones [7, 8]. In particular, the retardation of some projectiles in swine muscle has been shown to be comparable to that in human tissue [9–11]. Synthetic polymeric bone surrogates have also become popular in wound ballistic research with some authors claiming good representation of selected ballistic impacts on bone [e.g. 12–14]; however, others have observed that key features such as failure properties are not reproduced [e.g. 15, 16].

The majority of wound ballistics studies have been conducted with bullets rather than fragmentation. Fragmentation impacts onto isolated skin and soft tissue have been reported [e.g. 11, 17], but there is a paucity of literature considering fragmentation impacts onto isolated bone.

Testing of isolated bone requires the removal of soft tissue without altering the properties of the bone. Bacterial maceration is commonly discussed in the literature, sometimes due to the addition of a detergent or enzyme; in addition to this, chemical cleaning is also used, but there are concerns regarding alteration of bone properties [18–21]. There are many organisms including species of insect of the genus *Dermestes* (Dermestidae: Coleoptera) that consume flesh. Large museums often use carrion beetles of the family Dermestidae to clean bones [19]. These carnivorous beetles are 5–10 mm long as adults and 5–15 mm long in larval stages, thus are small enough to remove soft tissue between bones yet large enough to consume it quickly.

Bone fractures due to ballistic injury are clinically classified as being:

(i) ‘incomplete’—subdivided into ‘drill-hole’ in which the bone remains in one piece with a perforating hole and ‘chip type’ which is a penetrating impact, or
(ii) ‘complete’—sub-divided into ‘simple’ (two larger fragments are formed) and ‘multi-fragmentary’ [22].

Up to a certain loading rate, bone behaves as an elastic material [23]. However, bone is strain-rate sensitive, and in high velocity impacts, it behaves as a brittle material, failing almost instantaneously and can form secondary fragmentation [e.g. 24, 25].

The aim of the research summarised in this paper was to conduct ballistic testing using two types of fragment simulating projectiles (FSPs) over a range of velocities to determine the properties of swine rib bones.

## Methods

**Fig. 1:**
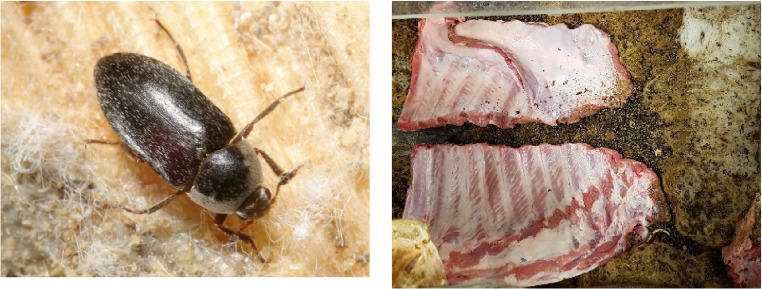
*Dermestes maculatus* (left) and swine thorax sections with beetles (right)

Four swine thorax sections were purchased from an abattoir where they had been prepared for use in the human food chain. The bone was stripped of all soft tissue by *Dermestes maculatus*, commonly called the Hide beetle as these were often identified as pests in museum skin collections (Fig. 1). The colony at The Natural History Museum is maintained in a purpose-built cabinet, under controlled conditions of temperature (23.2 to 28.5 °C), relative humidity (42.9 to 61.9% R.H.) and darkness. This method was selected as the beetles do not readily damage the bone unless left unchecked weeks after all the flesh has been consumed. After 3 weeks, all soft tissue had been consumed and the individual ribs were easily extracted. The ribs ranged in size from 70 to 210 mm long, 8 to 18 mm wide and 10 to 14 mm thick; each rib was given a unique identifier. The ribs were fumigated by freezing at − 4 °C for 24 h to prevent transference of live beetles and then defrosted over night at room temperature before being used in the ballistic tests. After testing, each rib was placed in a labelled bag and frozen until further analysis. Whether bone treated in this manner has exactly the same properties as fresh bone would have is not known, however, a study of fragment impacts into swine tissue that had been either refrigerated or frozen and then allowed to warm to room temperature reported similar results to fragment impacts into fresh swine tissue [26].

**Fig. 2:**
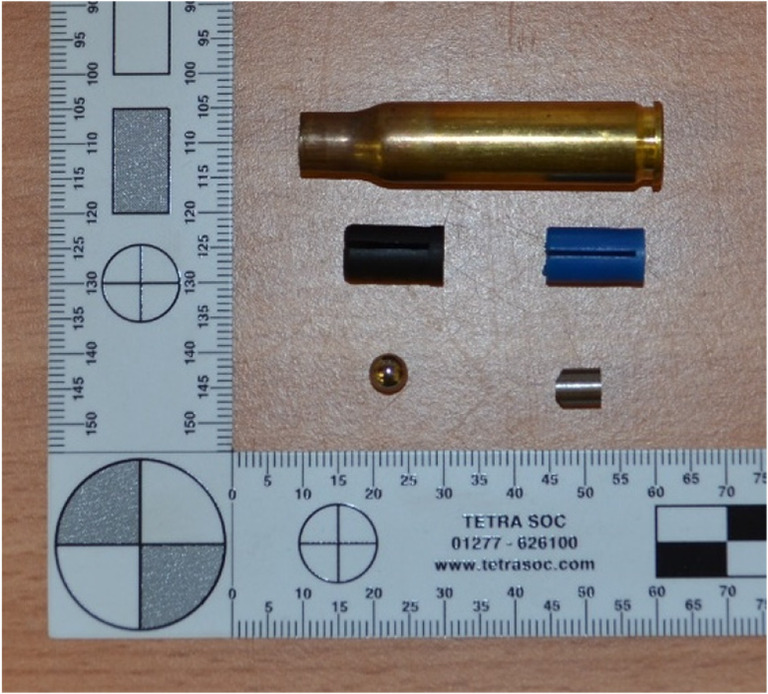
Cartridge case (top), sabots (middle), BB (bottom left) and CN FSP (bottom right)

The ballistic performance of the bone was measured using two types of internationally recognised steel fragment simulating projectiles (FSPs): (i) ball bearings (BBs; mass = 0.68 g) and (ii) chisel nosed FSPs (CN FSPs; mass = 1.10 g) (Fig. 2) [27]. Each FSP was placed in a sabot and then in a cartridge case. The impact velocity of the shot was adjusted by varying the amount of propellant placed in the cartridge case. The FSPs were fired from an Enfield Number 3 proof housing.

**Fig. 3:**
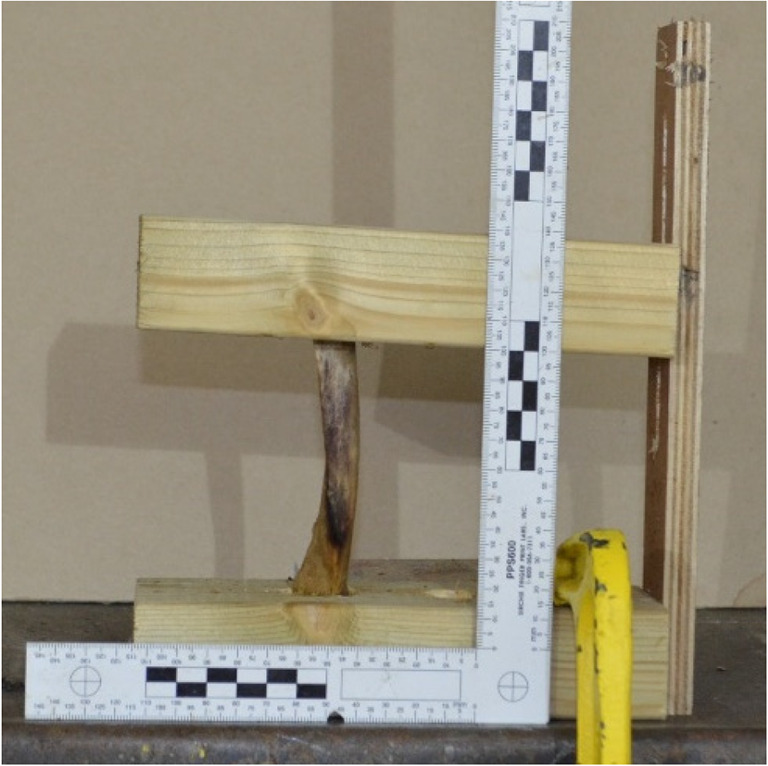
Custom-made wooden frame used to hold each rib specimen

Each rib was placed in a wooden frame that was clamped to a height adjustable table (Fig. 3). The proof housing was located 3 m from the test specimen, with the accuracy of each shot ensured via a laser sight mounted to the proof housing. Twenty ribs were shot with BBs and 15 ribs were shot with CN FSPs; each rib was subject to a single shot.

Post impact analysis determined whether or not each specimen was perforated by the projectile. Impact events were recorded using a Phantom Vision (V12) high-speed video (21,005 fps, 5 μs exposure time, 512 × 512 resolution) and these files were used to calculate the impact and residual velocities of the FSP after perforation of the rib. The video also allowed confirmation that the projectile was the cause of any perforation and not a secondary projectile such as the sabot. The impact and residual FSP velocities allowed the impact and residual kinetic energy (KE) of each FSP to be calculated (assuming conservation of mass). Subtracting the residual KE from the impact KE gave the energy dissipated during the impact event. In ballistics research, the target effects for projectiles with different projected areas are usually compared by considering the kinetic energy density of the projectile (KED; KE divided by project cross-sectional area, J/mm^2^). Each specimen was photographed before and after testing using a Nikon D90 digital camera with a forensic scale.

A Nikon Metrology X-TEK H225 micro-computed tomography (micro-CT) scanner with a tungsten transmission target was used to inspect selected specimens allowing sub-surface observation of the ballistic failures. Working conditions were as follows: 95 kV, 45 μA, 500 ms exposure time, two frames per projection, with an optimised number of projections; the resultant voxel size was 45.1 μm. Data was collected using Inspect-X software (v3.1.12), reconstructed using CT Pro 3D software (v. 3.1.12) and the images were visualised and manipulated with VG StudioMax software (v. 2.1). TIFF image stacks, with intervals of 0.05 mm between each slice, and whole volume reconstructions were generated.

## Results

Impact velocities varied between 99 and 1265 m/s. High speed footage and FSPs collected post-testing confirmed conservation of mass of the FSPs. Of the 35 shots, two were not on target (both were with CN FSPs at ~ 200 m/s); high-speed video showed that these two CN FSPs were not stable in flight. In both of these shots, the rib was hit by the sabot, and thus, another shot could not be taken for that rib. Ribs were perforated by all 33 FSP impacts that were on target. For shots at faster velocities (BBs > 700 m/s, KE > 170 J; CN FSPs > 580 m/s, KE > 110 J), four out of ten BB and six out of ten CN FSP impacts resulted in ribs fracturing into two parts, i.e. complete simple fractures, but with multiple small fractures being formed in all instances. At slower impact velocities, all ribs remained intact irrespective of FSP type, i.e. incomplete fractures with drill holes were observed.

**Fig. 4:**
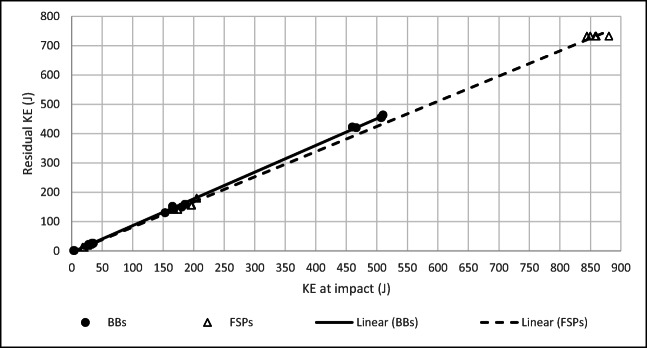
Relationship between impact and residual KE for both FSPs

The relationship between FSP KE at impact and residual KE was linear over the velocity regimes investigated for both types of FSP (*R*^2^ = 0.99 for both relationships; Fig. 4). However, the residual KE for BBs was greater than for CN FSPs at impact KEs greater than approximately 100 J.

**Fig. 5:**
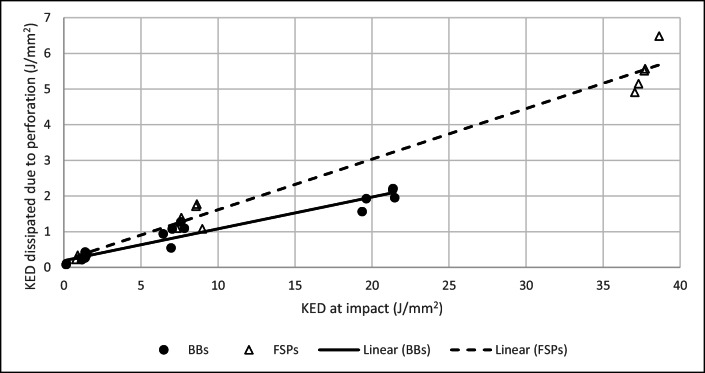
Relationship between KED at impact and the KED dissipated due to perforating event for both FSPs

As well as the mass of the two FSPs being different (BBs = 0.68 g, CN FSPs = 1.10 g), the projected cross-sectional area was different, 23.76 mm^2^ and 22.78 mm^2^ for the BBs and CN FSPs, respectively. The KED dissipated can be considered with respect to the KED at impact (Fig. 5). The KED dissipated during the impact event was greater for CN FSPs than for BBs and the magnitude of the difference increased with greater impact KED. For both FSPs, the relationship could be described as linear (CN FSP *R*^2^ = 0.98; BB *R*^2^ = 0.94).

**Fig. 6:**
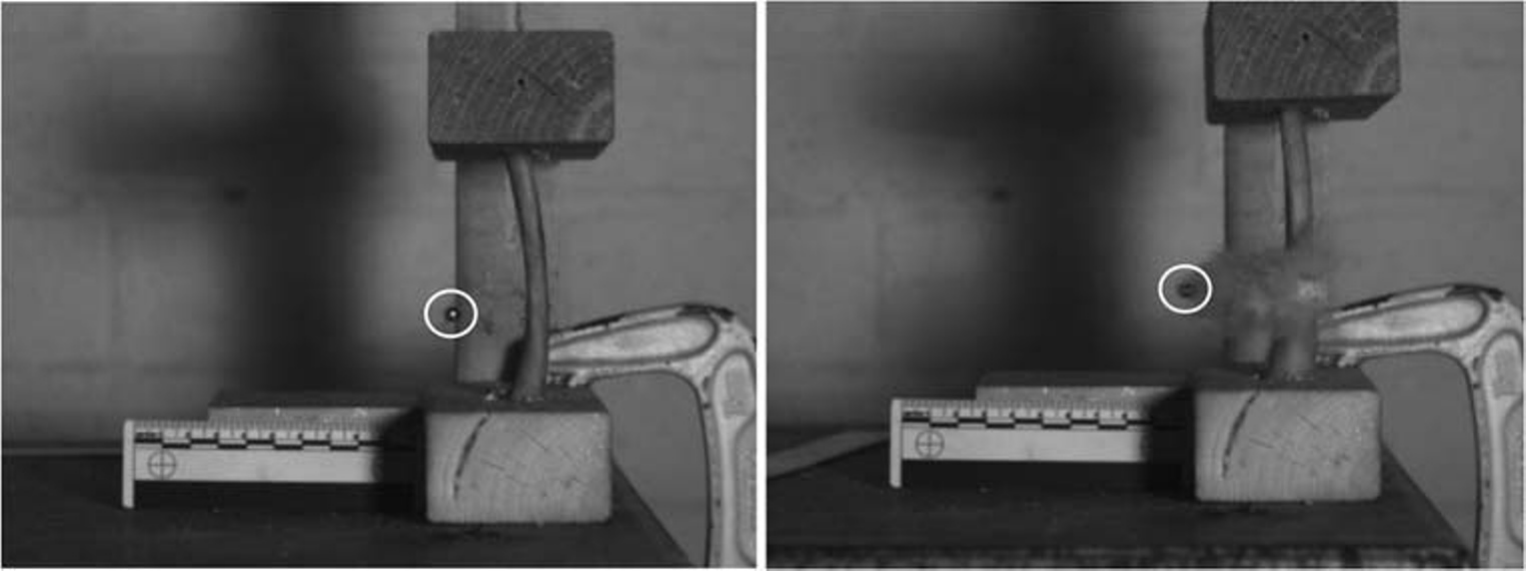
High speed video images demonstrating typical BB (circled) impacts perforating isolated swine ribs at 104 m/s (left) and 1221 m/s (right—note cloud of secondary fragments)

High speed video was used to analyse the response of the ribs to impact at different velocity regimes. For slower impact velocities (for BBs between 99 and 323 m/s; for CN FSPs between 180 and 217 m/s), the ribs displayed an initial deformation at the point of impact that was elastic; the curvature of the length of the rib changed during the impact event, but recovered. The rib then deformed plastically as failure occurred; as the dissipated energy at the point of the impact increased the rib failed (e.g. Fig. 6). Once the localised failure of the bone had occurred and the projectile had passed through the bone, elastic recovery occurred. In contrast, at faster velocities (BBs 671 m/s to 1225 m/s; CN FSPs 551 m/s to 1265 m/s), no elastic deformation was observed. Instantaneous failure occurred at the point of impact, with multiple secondary fragments exiting both the anterior and the posterior of the specimen, primarily the latter (Fig. 6).

Of the ribs that were scanned, two were of particular interest as the damage caused by both types of FSPs exhibited similar amounts of KED dissipated during perforation:

(i) BB impact velocity 671 m/s, KED dissipated 0.94 J/mm^2^
(ii) CN FSP impact velocity 610 m/s, KED dissipated 1.08 J/mm^2^

**Fig. 7:**
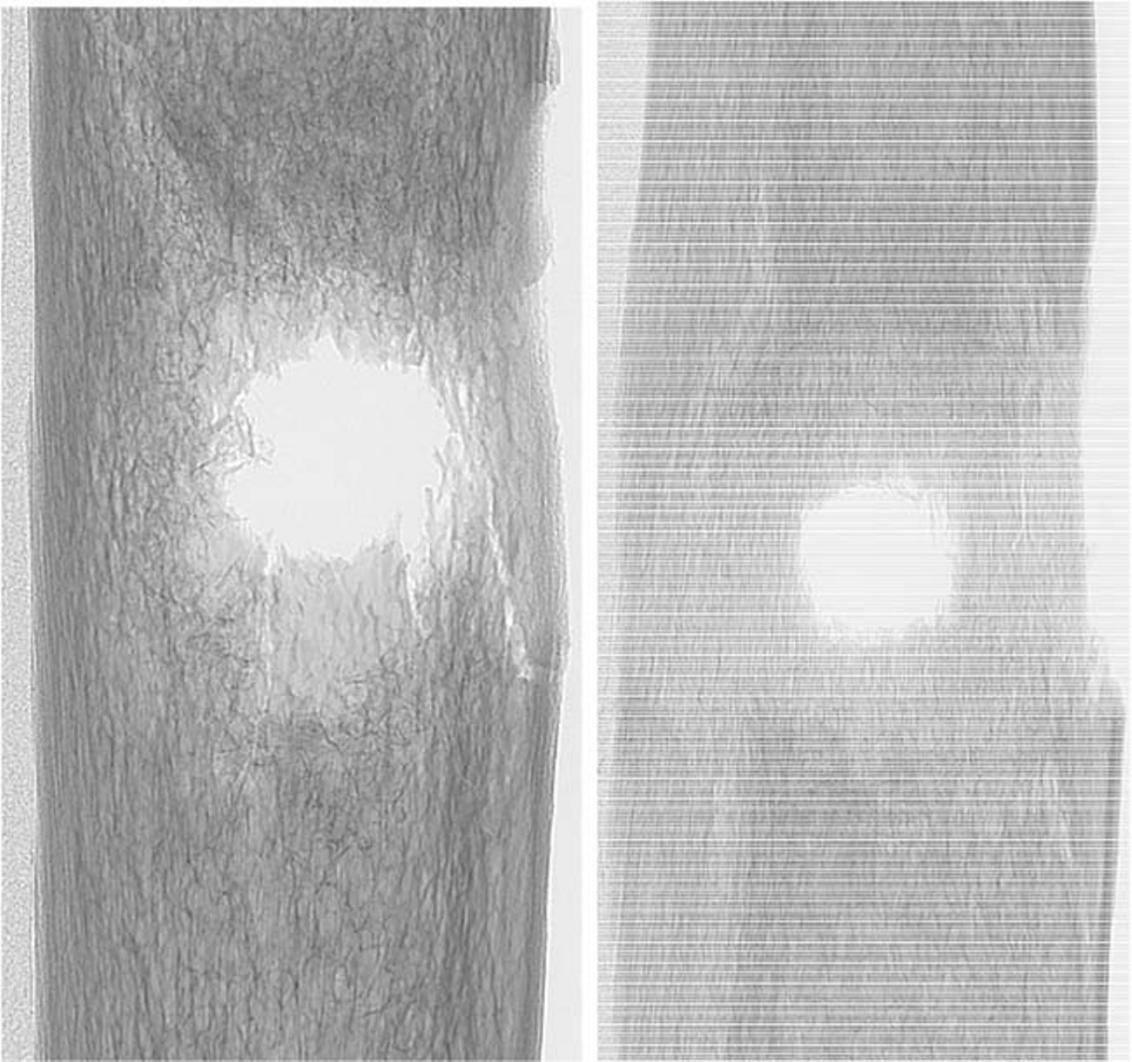
X-radiographs of specimens after a perforating impact from a BB at 671 m/s (left) and a CN FSP at 610 m/s (right)

**Fig. 8:**
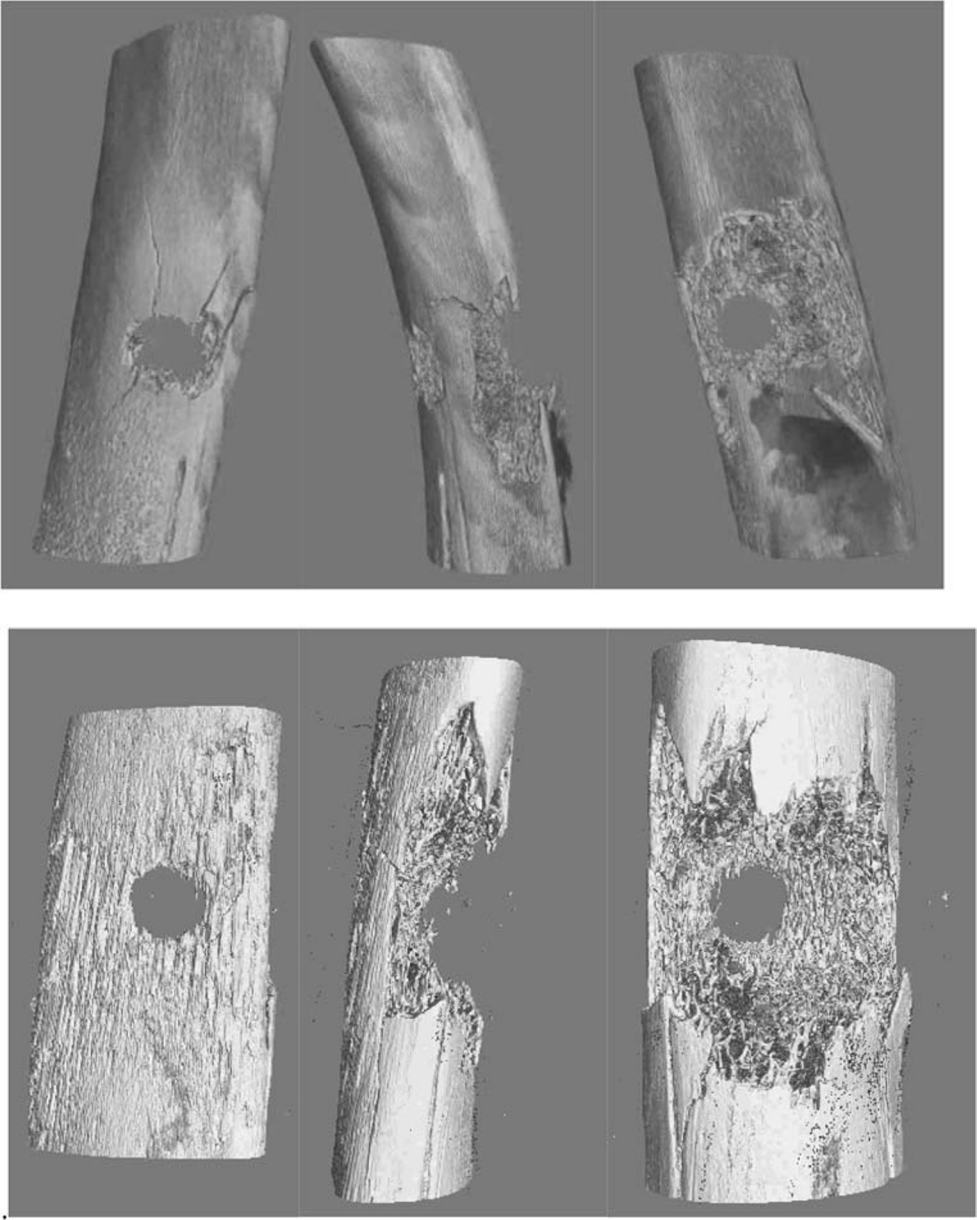
Three-dimensional volume reconstruction of micro-CT data of a specimens after a perforating impact from a BB at 671 m/s (upper set of images) and a CN FSP at 610 m/s (lower set of images); for both sets of images: left = anterior; centre = side; right = posterior

X-radiographs of these two impacts are shown in Fig. 7 and three-dimensional volume reconstructions of micro-CT data in Fig. 8. Comparing these images, the loss of material appeared to be larger for the CN FSP compared to the BB at a similar impact velocity. The CN FSP was heavier than the BB (1.1 g vs 0.68 g) resulting in a larger KE at impact. The projected cross-section area of the BB was slightly larger than that of the CN FSP (CN FSP = 22.78 mm^2^; BB = 23.76 mm^2^), thus affecting the KED at impact. This result may therefore be due to the irregular shape of the CN FSP and how it moves inside the bone after impact. The greater loss of materials observed for the CN FSP impact may also be due to yaw during perforation.

## Discussion

The aim of this research was to investigate the ballistic performance of bone following impacts from two internationally recognised FSPs. All impacts on target perforated the bone. Two CN FSPs did not impact the target and these FSPs yawed during bone perforation—an advantage of using BBs is that yaw does not occur. The slowest impact velocity, with perforation, was 99 m/s. This aligns with a previously reported threshold for perforation of human bone of ~ 61 m/s, but the authors did not state which projectile was used [22]. Slower impact velocities resulted in localised bone damage, i.e. incomplete drill-hole fractures. At faster impact velocities, greater bone fragmentation occurred and half of the impacts resulted in complete fractures; previous authors have described similar responses [e.g. 22, 23, 25]. The primary projectile (FSPs in the current experiment) remains the major threat. However, there is potential for these bone fragments to act as secondary fragments that may penetrate (and perforate) the organs of the thorax. Mabbott et al. reported bone fragment debris in isolated lungs positioned behind swine thorax sections due to perforation by selected bullets [24]. In a living target, aspiration may further contribute towards the presence of bony fragments (and other debris) in lung tissue.

The conical damage observed on the posterior side of the ribs is similar to that reported by Kieser et al. [25]. Energy transfer to the bone was greater for the CN FSPs, resulting in increased bone damage and the production of secondary bone fragments. Such additional fragments may risk damage to vascular structures, which often run in close proximity to bones, and may result in internal bleeding. The differences in failure mechanisms for the different types of FSPs have clinical implications and do not appear to have been reported previously.

## Conclusion

This study gives critical insight into how isolated bone behaves under ballistic impact. Information is provided for the KED dissipated during bone perforation for velocities between 99 and 1265 m/s, using two internationally recognised FSPs. Energy transfer to the bone was greater for the CN FSPs, resulting in increased bone damage and the production of secondary bone fragments. Such additional fragments may risk damage to vascular structures, which often run in close proximity to bones, and may result in internal bleeding.

## Limitations

The results in this paper are for ballistic impacts to isolated swine ribs using two specific FSPs; results may vary for other projectiles. Although swine tissue is an accepted model for human tissue, the results may not be transferable. Finally, the isolated bony tissue used was effectively 3 weeks old at testing; fresh bony tissue might behave in a different manner.

## References

[1] Keene DD, Penn-Barwell JG, Wood PR, Hunt N, Delaney R, Clasper J, Russell RJ, Mahoney PF (2015). Died of wounds: a mortality review. J R Army Med Corps.

[2] Karger B, Zweihoff RF, DuChesne A (1999). Injuries from hand grenades in civilian settings. Int J Legal Med.

[3] Lewis EA (2006) Between Iraq and a hard plate: recent developments in UK military personal armour. In: IPAC, ed. Personal Armour Systems Symposium (PASS 2006) The Royal Armouries, Leeds

[4] Spalding TJW, Stewart MPM, Tulloch DN, Stephens KM (1991). Penetrating missile injuries in the Gulf War 1991. BJS Open.

[5] Humphrey C, Kumaratilake J (2016). Ballistics and anatomical modelling – a review. Legal Med.

[6] Carr DJ, Stevenson T, Mahoney PF (2018). The use of gelatine in wound ballistics research. Int J Legal Med.

[7] Carr DJ, Kieser J, Mabbott AJ, Mott C, Champion S, Girvan E (2014). Damage to apparel layers and underlying tissue due to hand-gun bullets. Int J Legal Med.

[8] Cohen H, Kugel C, May H, Medlej B, Stein D, Slon V, Hershkovitz I, Brosh T (2016). The impact velocity and bone fracture pattern: forensic perspective. Forensic Sci Int.

[9] Wilson LB (1921) Dispersion of bullet energy in relation to wound effects. The Military Surgeon XLIX: 241–51

[10] Fackler ML, Surinchak JS, Malinowski JA, Bowen RE (1984). Wounding potential of the Russian AK-74 assault rifle. J Trauma.

[11] Breeze J, Hunt NC, Gibb I, James GR, Hepper AE, Clasper JC (2013). Experimental penetration of fragment simulating projectiles into porcine tissues compared with simulants. J Forensic Legal Med.

[12] Mahoney PF, Carr DJ, Delaney RJ, Hunt N, Harrison S, Breeze J, Gibb I (2017). Does preliminary optimisation of an anatomically correct skull-brain model using simple simulants produce clinically realistic ballistic injury fracture patterns?. Int J Legal Med.

[13] Smith MJ, James S, Pover T, Ball N, Barnetson V, Foster B, Guy C, Rickman J, Walton V (2015). Fantastic plastic? Experimental evaluation of polyurethane bone substitutes as proxies for human bone in trauma simulations. Legal Med.

[14] Kneubuehl BP, Thali MJ (2003). The evaluation of a synthetic long bone structure as a substitute for human tissue in gunshot experiments. Forensic Sci Int.

[15] Bir C, Andrecovich C, DeMaio M, Dougherty PJ (2016). Evaluation of bone surrogates for indirect and direct ballistic fractures. Forensic Sci Int.

[16] Mahoney PF, Carr DJ, Hunt N, Delaney RJ (2019). Assessment of polyurethane spheres as surrogates for military ballistic head injury. Int J Legal Med.

[17] Breeze J, James GR, Hepper AE (2013). Perforation of fragment simulating projectiles into goat skin and muscle. J R Army Med Corps.

[18] Simmons J, Muñoz-Saba Y (2005) Esqueletos. In: Simmons J, Muñoz-Saba Y (eds) Cuidado, Manejo y Conservación de las Colecciones Biológicas. Bogotá D.C., Colombia: Conservación Internacional. Serie Manuales para la Conservación, No. 1, Instituto de Ciencias Naturales, Facultad de Ciencias, Universidad Nacional de Colombia, Conservación Internacional Colombia, pp 91–125

[19] Brito de Oliveira M (2018). Methods of biological maceration in the preparation of bat skulls: benefits and limitations. Pap Avulsos Zool.

[20] Sullivan L (1999). Cleaning and preserving animal skulls.

[21] Fragkouli K, Al Hakeem E, Bulut O, Simmons T (2018). The effect of range and ammunition type on fracture patterns in porcine postcranial flat bones. J Forensic Legal Med.

[22] Griffiths D, Clasper J (2006). Military limb injuries/ballistic fractures. Curr Orthop.

[23] Paschall A, Ross AH (2017). Bone mineral density and wounding capacity of handguns: implications for estimation of caliber. Int J Legal Med.

[24] Mabbott A, Carr DJ, Champion S, Malbon C (2014) Boney debris ingress into lungs due to gunshot. International Symposium on Ballistics Atlanta, USA

[25] Kieser DC, Riddell R, Kieser JA, Theis JC, Swain MV (2013). Bone micro-fracture observations from direct impact of slow velocity projectiles. J Arch Mil Med.

[26] Breeze J, Carr DJ, Mabbott A, Beckett S, Clasper JC (2015). Refrigeration and freezing of porcine tissue does not affect the retardation of fragment simulating projectiles. J Forensic Legal Med.

[27] The NATO Standardization Office (2015) NATO Standard AEP-2920 Procedures for the evaluating and classification of personal Armour bullet and fragmentation threats Edition A Version 1. Brussels

